# Case Report: A case of progressive encephalopathy with or without lipodystrophy caused by BSCL2 variant and literature review

**DOI:** 10.3389/fgene.2025.1528563

**Published:** 2025-02-28

**Authors:** Yao Wang, Jing Guo, Peiqi Zhang, Fang Liu, Hua Li

**Affiliations:** ^1^ Department of Neurology, Guangdong Sanjiu Brain Hospital, Guangzhou, China; ^2^ Department of Pediatric Neurology, Guangdong Women and Children Hospital, Guangzhou, China

**Keywords:** BSCL2 gene, progressive encephalopathy with or without lipodystrophy, celia’s encephalopathy, progressive myoclonus epilepsy, neurological regression

## Abstract

**Objectives:**

To describe a case of Progressive Encephalopathy with or without Lipodystrophy (PELD), characterized by a late onset of neurological regression at 9 years old, due to a homozygous c.974dupG variant in the BSCL2 gene.

**Methods:**

An 11-year, 9-month-old girl with repeated seizures over 2 years underwent clinical assessment and genetic investigation. We also reviewed the published literature.

**Results:**

The patient exhibited mild intellectual disability, a lipodystrophic appearance, precocious puberty, voracious appetite, elevated transaminase levels, hyperlipidemia, hypercortisolism, hepatomegaly, fatty liver, and splenomegaly. Motor and cognitive regression occurred at 9 years. A homozygous pathogenic variant c.974dup (p.Ile326HisfsTer12) in exon 7 of BSCL2 (NM_001122955.4) was identified. Despite multiple antiseizure medications, seizures were refractory, leading to status epilepticus and rapid death after genetic diagnosis.

**Conclusion:**

We confirm that the BSCL2 c.974dupG variant is a cause of PELD. Regression may occur later than previously reported. Literature review suggests that the c.974dupG variant may present a milder phenotype compared to the classic c.985C>T variant. Early genetic testing and diagnosis are crucial for improving outcomes in rare neurodegenerative disorders like PELD.

## Introduction

The BSCL2 gene is located on the long arm of chromosome 11 at position 11q13. It encodes the protein seipin, an integral membrane protein of the endoplasmic reticulum (ER). The gene is highly expressed in the brain (Magré et al., 2001), encoding three main seipin isoforms: 462 (BSCL2-203), 398 (BSCL2-205/207/210) and 287 (BSCL2-201) amino acids long, respectively (Guillén-Navarro et al., 2013). Biallelic mutation in the BSCL2 gene can cause progressive encephalopathy with or without lipodystrophy (PELD; MIM: #615924) and congenital generalized lipodystrophy type 2 (CGL2; MIM: #269700). Heterozygous mutations in the BSCL2 gene can cause distal hereditary motor neuronopathy type VC(HMND13; MIM: #619112) and Silver syndrome (SPG17; MIM: #270685). PELD, also known as Celia’s encephalopathy, is a rare and severe neurodegenerative disorder. It is characterized by developmental regression of motor and cognitive skills before the age of 5, often leading to death within the first decade. Patients may exhibit a mild or typical lipodystrophic appearance (Guillén-Navarro et al., 2013). The variant c.985C>T is considered the classic genotype (Guillén-Navarro et al., 2013). However, other variants in BSCL2 associated with severe neurodegenerative manifestations have been reported, some of which resemble PELD. We report a patient with PELD due to a homozygous c. 974dupG variant in the BSCL2 gene, who experienced motor and cognitive regression at 9 years old. We also review the features of all published PELD patients.

## Methods

The study was approved by the ethics review committee of Guangdong Sanjiu Brain Hospital and conducted according to the Helsinki Declaration’s ethical guidelines. Consent for disclosure was obtained from the patient’s parents. We also reviewed published literature.

## Results

### Case description

**FIGURE 1:**
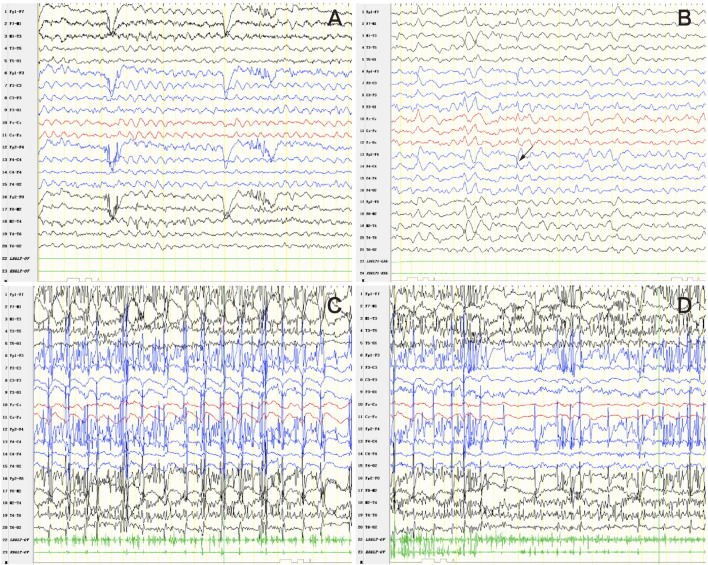
VEEG findings of the PELD patient: **(A)** Slow background; **(B)** Diffuse epileptiform discharges (indicated by the arrow); **(C)** Rhythmic myoclonus; **(D)** Irregular myoclonus (action myoclonus).

**FIGURE 2:**
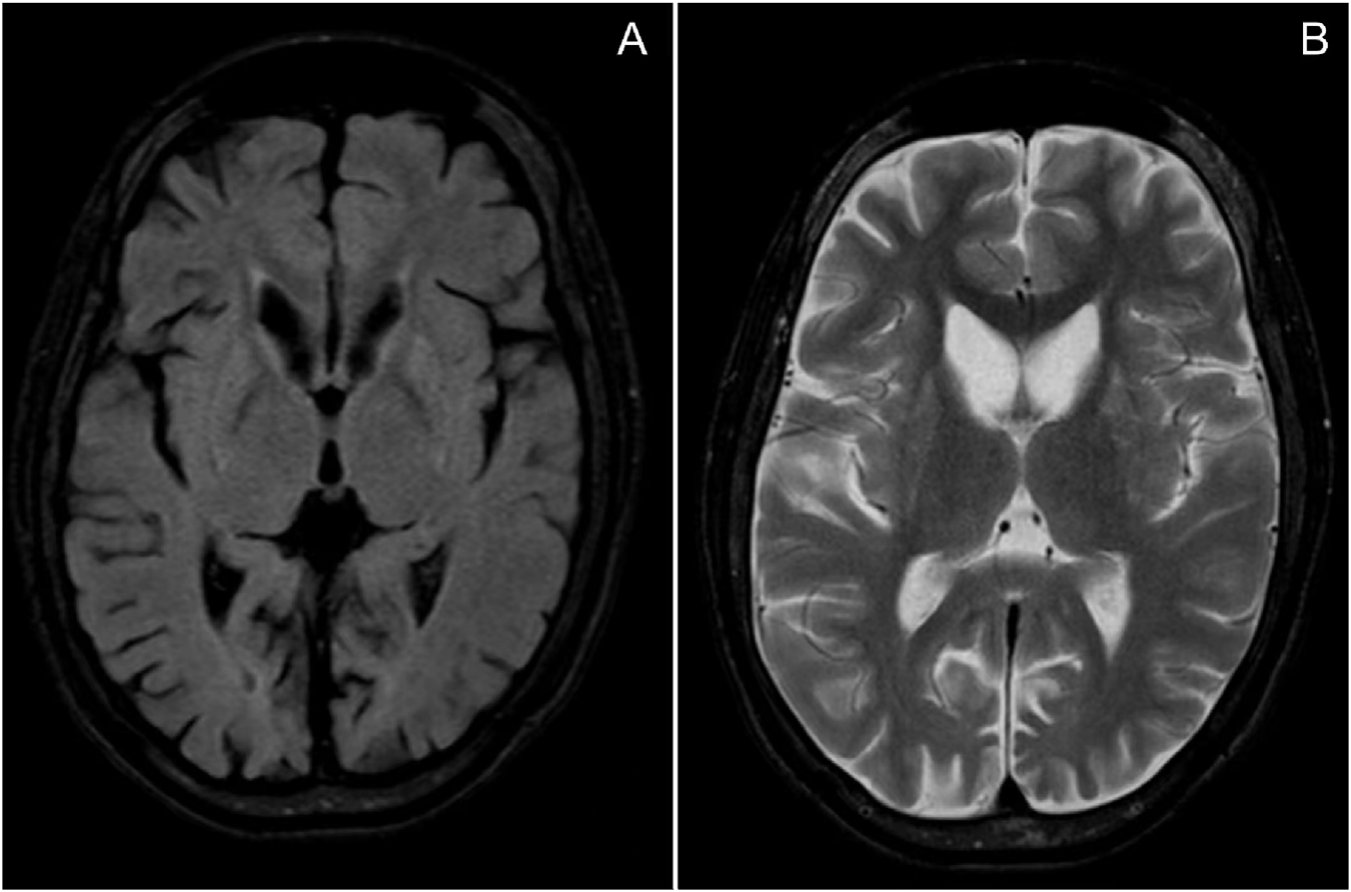
MRI of the patient: **(A)** T2 FLAIR; **(B)** T2-weighted imaging (T2WI), indicating diffuse brain atrophy, particularly in the caudate nucleus.

An 11-year, 9-month-old girl was hospitalized in June 2024, due to repeated seizures over 2 years. She was born to non-consanguineous parents in Guangdong Province, China. The pregnancy and delivery were uneventful. Mild intellectual disability was noted at 30 months. In infancy, she exhibited a lipodystrophic appearance and acanthosis nigricans. She experienced precocious puberty, voracious appetite, elevated transaminase levels, hyperlipidemia, hypercortisolism, hepatomegaly, fatty liver, and splenomegaly. At 9 years old, the patient experienced epileptic seizures characterized by generalized tonic-clonic seizures (GTCS) and myoclonus. The seizures were refractory despite treatment with levetiracetam and topiramate. She experienced a status epilepticus (SE) episode lasting approximately 10 h. At that time, the patient demonstrated psychomotor regression. She exhibited motor deterioration, ataxia, action myoclonus, tremors, reduced speech, dysarthria, and sleep disturbances. By 11 years and 9 months, the patient could only speak single words and had no independent gait, requiring assistance for eating, bathing, and toileting. Physical examination revealed marked generalized lipoatrophy, hypotonia, ataxia, and action myoclonus. Video electroencephalography (VEEG) indicated a slow background and diffuse epileptiform discharges. Frequent myoclonus was recorded (Figure 1). Brain MRI indicated diffuse brain atrophy, particularly in the caudate nucleus (Figure 2). Gynecological ultrasound indicated a small uterine volume. Blood glucose, lipids, lactic acid, urine organic acids, electrocardiogram, echocardiogram, and abdominal ultrasound were unremarkable. After obtaining informed consent from the parents, whole exome sequencing was conducted. Genetic analysis revealed a homozygous pathogenic frameshift variant in BSCL2 (NM_001122955.4:c.974dup; p.Ile326HisfsTer12), classified as pathogenic according to ACMG guidelines, with both parents identified as heterozygous carriers. Clonazepam and perampanel were successively prescribed, providing transient seizure control. Seizure occurrences gradually increased and progressed to SE on July 23rd. She died at the age of 11 years and 11 months from SE on July 25th.

### Review of previous cases

Clinical, imaging and genetic findings of our patient and the other 23 reported PELD patients are summarized in Tables 1, 2 (Guillén-Navarro et al., 2013; Alaei et al., 2016; Zhang et al., 2019; Wu et al., 2009; Sánchez-Iglesias et al., 2019; Fernández-Marmiesse et al., 2019; Opri et al., 2016; Ferranti et al., 2020; Serino et al., 2019; Pedicelli et al., 2020; Araújo-Vilar et al., 2018; Poisson et al., 2019; Stanley et al., 2022; Ding et al., 2021).

**TABLE 1:** Clinical features of PELD patients (part 1).

Case	Variant	Genotype	Nation	Sex	Age of neurological symptoms onset	Age of regression	Neurological symptoms	Seizure type	Lipodystrophic appearance	Reference
1	985C>T; 538G>T	Compound heterozygote	Spain	M	3 years	4 years	Hyperactivity, cognitive impairment, language delay, tremor, seizures, dystonia, dysphagia	Myoclonus, focal and generalised seizures	Y	Guillén-Navarro et al. (2013)
2	985C>T; 509_513del	Compound heterozygote	Spain	F	2 years	N. R.	Developmental delay, neurological regression, seizures	Myoclonus	Y	Araújo-Vilar et al., 2018
3	985C>T; 507_511del	Compound heterozygote	Spain	M	2 years	5 years	Abnormal communication skills, language delay, hyperactivity, seizures, cognitive impairment, ataxic gait	Myoclonus, focal seizures, GTCS	Y	Guillén-Navarro et al. (2013)
4	985C>T; 507_511del	Compound heterozygote	Spain	M	3 years	N. R.	Hyperactivity, language delay, seizures	Myoclonus	Y	Guillén-Navarro et al. (2013)
5	985C>T; 507_511del	Compound heterozygote	Spain	F	3.5 years	no regression	Psychomotor delay	None	Y	Guillén-Navarro et al. (2013)
6	985C>T; 1004A>C	Compound heterozygote	Spain	F	3 years	N. R. (before 6y)	Anxiety, irritability, strabismus, dystonic hypertonia, extrapyramidal and pyramidal features, dysphagia, parkinsonism, frontal lobe syndrome, dementia	None	N	Poisson et al. (2019)
7	985C>T	Homozygote	Spain	F	2 years	3 years	Cognitive impairment, ataxic gait, tremor, dystonia, sleep disturbances, seizures, tetraparesis	GTCS, myoclonus	N	Guillén-Navarro et al. (2013)
8	985C>T	Homozygote	Spain	F	before 19 months	5 years	Developmental delay, irritability, dysphagia, sleep disturbances, pyramidal signs	Myoclonus	N	Guillén-Navarro et al. (2013)
9	985C>T	Homozygote	Iranian	M	2 years	N. R.	Hyperactivity, autistic, ataxia, generalized hypertonia, global developmental delay	NCSE, convulsive epilepsy	Y	Alaei et al. (2016)
10	974dupG; 757G>T	Compound heterozygote	China	M	N. R.	no regression	Dystonia, mild intellectual disability	None	Y	Wu et al. (2009)
11	974dupG; 1020_1021delAA	Compound heterozygote	Italy	M	1 years	5 years	Developmental delay, ataxic gait, intention myoclonus, pyramidal signs, loss of language, intellectual impairment, dystonic tetraplegia	Absence seizures with myoclonia or eyelid myoclonia, myoclonus	Y	Opri et al. (2016)
12	974dupG; 1015C>T	Compound heterozygote	Spain	F	2 years	no regression	Speech delay	None	Y	Sánchez-Iglesias et al. (2019)
13	974dupG	Homozygote	China	M	1–2 years	5 years	Psychomotor delay, hyperactivity, irritability, unstable walking, tremor, dysarthria, cognitive regression	Absence seizures, atonic seizures, myoclonic-atonic seizures, myoclonus	Y	Ding et al. (2021)
14	974dupG	Homozygote	China	M	3 years	15 years	Dystonic hypertonia, involuntary shaking of the body, opisthotonus, seizures	Myoclonus	Y	Zhang et al. (2019)
15	974dupG	Homozygote	Italy	F	3 years	6 years	Intellectual disability, continuous erratic myoclonias, pyramidal signs, cognitive decline, tetraparetic	Absence seizures with eyelid myoclonia, myoclonic-atonic seizures	Y	Opri et al. (2016)
16	974dupG	Homozygote	Italy	F	N. R. (early childhood)	5 years	Intellectual disability, cognitive decline, progressively increasing myoclonus, pyramidal signs, tetraparesis, intellectual disability	Absence seizures with eyelid myoclonia, myoclonic-atonic seizures	Y	Opri et al. (2016)
17	974dupG	Homozygote	Spain	F	18 months	6 years	Language delay, stereotyped movements, progressive motor, language, and cognitive deterioration, dysphagia, seizures	Myoclonus, GTCS	Y	Sánchez-Iglesias et al. (2019)
18	974dupG	Homozygote	China	F	30 months	9 years	Seizures, developmental delay, language and motor regression, tremor, sleep disturbances, ataxic gait, myoclonus	GTCS, myoclonus	Y	Our case
19	566T>A	Heterozygote	Spain	M	6 months	N. R.	Seizures, intellectual disability, autism spectrum disorder, ataxic gait	GTCS, absences with eyelid myoclonia, atonic seizures	N	Fernández-Marmiesse et al. (2019)
20	566T>A	Heterozygote	Spain	F	3 months	8 months	Seizures, psychomotor delay	Spasms, SE	N	Fernández-Marmiesse et al. (2019)
21	1048C>T	Homozygote	Italy	F	N. R.	5 years	Language delay, irritability, psychomotor agitation, progressive language impairment, bulbar signs, lost gait	Myoclonus, sGTCS	Y	Ferranti et al. (2020)
22	1048C>T	Homozygote	Italy	F	N.R.	5 years	Language delay, irritability, psychomotor agitation, progressive language impairment, bulbar signs, lost gait	Myoclonus, sGTCS	Y	Ferranti et al. (2020)
23	445C>G	Heterozygote	USA	M	2 weeks	7 years	Seizures	Spasms, SE	N	Stanley et al. (2022)
24	1076dupC	Homozygote	Macedoniay	M	1 years	3 years	Psychomotor delay, hyperactivity, ataxic gate, seizures	Generalized tonic seizures, absence seizures with eyelid myoclonia, NCSE	Y	Serino et al. (2019), Pedicelli et al. (2020)

F: female. M: male. GTCS: generalized tonic-clonic seizures. SE: status epilepticus. NCSE: Non convulsive status epilepticus. Y: Yes. N: No. N. R.: Not reported.

**TABLE 2:** Clinical features of PELD patients (part 2).

Case	Variant	Other non-neurological symptoms	Treatment	Age of decease (cause) or Age of last follow-up	Brain MR	EEG	Reference
1	985C>T; 538G>T	None	N. R.	8 years (SE)	Normal	Generalised spike	Guillén-Navarro et al. (2013)
2	985C>T; 509_513del	Hepatomegaly, hypertriglyceridemia, hyperinsulinemia, hypertransaminasemia, low plasma leptin levels	Metreleptin, diet rich in PUFA, omega-3 fatty acid supplementation, ASMs	survival at 7 years 10 months	Atrophy of caudate nuclei, thalami and outer capsules, hypersignal of periventricular white matter	Frequent bursts of spike activity at both central-parieto-temporo-occipital lobes	Araújo-Vilar et al., 2018
3	985C>T; 507_511del	None	N. R.	7 years (respiratory infection)	N. R.	N. R.	Guillén-Navarro et al. (2013)
4	985C>T; 507_511del	Hepatomegaly, hypertriglyceridaemia	N. R.	7 years (respiratory infection)	N. R.	N. R.	Guillén-Navarro et al. (2013)
5	985C>T; 507_511del	None	N. R.	survival at 11 years	N. R.	N. R.	Guillén-Navarro et al. (2013)
6	985C>T; 1004A>C	None	levodopa	28 years (pneumonia)	Caudate nucleus atrophy	Normal	Poisson et al. (2019)
7	985C>T	Hepatomegaly, hypertriglyceridaemia, coarse facies, striking muscle induration of the limbs	N. R.	8 years (aspiration pneumonia)	Subcortical atrophy	Multifocal and sporadic generalised spike	Guillén-Navarro et al. (2013)
8	985C>T	None	N. R.	6 years (N. R.)	N. R.	N. R.	Guillén-Navarro et al. (2013)
9	985C>T	Hypertrichosis	ASMs	8 years (SE)	An arachnoid cyst in the left hippocampus	NCSE	Alaei et al. (2016)
10	974dupG; 757G>T	Diabetes, hypertriglyceride, fatty liver, splenomegaly	N. R.	survival at 28 years	Normal	N. R.	Wu et al. (2009)
11	974dupG; 1020_1021delAA	Feeding problems, reduced alertness, hypertriglyceridemia, hypertransaminasemia, hepatic steatosis, hypertrophic cardiomyopathy	low-fat diet, ASMs	9 years 10 months (cachexia)	Progressive cortico-subcortical cerebral atrophy, particularly involving the caudate head and the lenticulate nucleus, hypersignal of subcortical structures and periventricular white matter	Theta activity and diffuse spike; Background activity deterioration, nearly continuous myoclonic, photoparoxysmal response	Opri et al. (2016)
12	974dupG; 1015C>T	Failure-to-thrive, hypertriglyceridemia, liver steatosis	Metreleptin	survival at 2 years	N. R.	N. R.	Sánchez-Iglesias et al. (2019)
13	974dupG	Hepatomegaly, hepatic Insufficiency, insulin resistance	Low fat and low glycemic index diet, hypoglycemic drugs, ASMs	survival at 5 years 8 months	N. R.	Multifocal and generalized spike	Ding et al. (2021)
14	974dupG	Unable to feed	ASMs	survival at 17 years	Cerebral atrophy, basal ganglia atrophy	Focal epileptiform discharges	Zhang et al. (2019)
15	974dupG	Hepatomegaly, skin hyperpigmentation, reduced alertness, hypertriglyceridemia, cardiomegaly	N. R.	7 years 9 months (N. R.)	Enlarged ventricles and liquoral spaces (pneumoencephalography)	Multifocal or diffuse spike	Opri et al. (2016)
16	974dupG	Hypertriglyceridemia	N. R.	11 years 10 months (N. R.)	Diffuse cerebral atrophy, with marked involvement of caudate and lenticular nuclei	Background activity deterioration, increasing action myoclonus	Opri et al. (2016)
17	974dupG	Hepatomegaly, hypertriglyceridemia, feeding difficulties, hyporeactive, somnolent	ASMs	9 years 9 months (N. R.)	Progressive atrophy of striated regions	Diffuse theta and delta waves and low-voltage beta rhythm and multifocal epileptiform anomalies	Sánchez-Iglesias et al. (2019)
18	974dupG	Hypertriglyceridaemia, splenomegaly, fatty liver	ASMs	11 years (SE)	Cerebral atrophy	Slow background, focal and diffuse spike	Our case
19	566T>A	None	Ketogenic diet, ASMs	survival at 10 years	Normal	Slow background, focal and generalized spike	Fernández-Marmiesse et al. (2019)
20	566T>A	None	ASMs	10 months (SE)	Normal	Slow background, multifocal epileptiform activity	Fernández-Marmiesse et al. (2019)
21	1048C>T	Coarse facial features, synophrys, bulbous nasal tip, large ear pinnae, wide mouth, long fingers and toes, and hypertrichosis	ASMs	survival at 15 years	Hyperintensity and shrinkage of the putamen and caudate head, ventricular system enlargement	Slow background, multifocal and diffuse spike, cortical myoclonia	Ferranti et al. (2020)
22	1048C>T	Coarse facial features, synophrys, bulbous nasal tip, large ear pinnae, wide mouth, long fingers and toes, and hypertrichosis	ASMs	18 years (N. R.)	Hyperintensity and shrinkage of the putamen and caudate head, ventricular system enlargement	Slow background, multifocal and diffuse spike, cortical myoclonia	Ferranti et al. (2020)
23	445C>G	None	ASMs	7 months (N. R.)	Normal	Slowed and disorganized background, multifocal and generalized spike	Stanley et al. (2022)
24	1076dupC	Hypertriglyceridaemia, hypertransaminasaemia	Metreleptin, low-fat diet, ASMs, VNS	survival at 7 years	Normal	Abnormal background activity, photosensitivity, diffuse origin during ictal period	Serino et al. (2019), Pedicelli et al. (2020)

ASMs: antiseizure medications. SE: status epilepticus. N. R.: Not reported.

The patient cohort consisted of 11 males and 13 females, with cases distributed as follows: 12 from Spain, 5 from Italy, 4 from China, 1 from the United States, 1 from Iran, and 1 from Macedonia. 79.2% (19/24) of patients had neurological symptoms before the age of 3. (87.5%) 21 of cases experienced regression, with 14 regressing before the age of 6. 75% (18/24) of cases exhibited a lipodystrophic appearance. The cohort included homozygous (12/24), compound heterozygous (9/24), and heterozygous cases (3/24), with the c.985C>T and c.974dupG variants being the most common mutations. 15 patients died prematurely, with 11 dying before the age of 10. Nine patients were alive, with the oldest reported being 28 years old.

## Discussion

This study reports a girl with PELD caused by a c.974dupG biallelic mutation in the BSCL2 gene. A literature review revealed no gender differences among individuals with PELD. These cases were commonly reported in European and Asian countries. Most patients exhibited neurological symptoms before the age of 3 and experienced regression before the age of 6. Developmental delay and neurological regression were the most common neurological symptoms. The prognosis was poor, with most patients dying prematurely. Typical imaging showed brain atrophy, primarily in the caudate nucleus, and EEG findings indicated a slow background with multifocal or generalized epileptiform discharges.

Our case presents unique features including late-onset regression at 9 years old, rapid progression to death within 2 years after regression, and the presence of both lipodystrophic appearance and PME symptoms, which distinguishes it from previously reported cases. Among the documented cases, a notable c.974dupG mutation carrier manifested neurological regression at 15 years of age, representing the latest reported onset in the literature (Zhang et al., 2019). Remarkably, the longest documented survival was observed in a 28-year-old patient harboring the same mutation (Wu et al., 2009). These clinical observations collectively suggest that individuals with c.974dupG mutation exhibit delayed disease progression and extended survival compared to c.985C>T mutation carriers, potentially reflecting the differential impact of these variants on BSCL2 function.

The pathogenesis of Celia’s encephalopathy involves abnormal splicing of BSCL2 gene, leading to exon 7 skipping. This results in increased expression of BSCL2-201 transcript, which generates truncated seipin protein. The accumulation of aberrant seipin induces endoplasmic reticulum (ER) stress and the formation of intranuclear aggregates, ultimately triggering neuronal apoptosis (Sánchez-Iglesias et al., 2021). Notably, patients with c.974dupG mutation exhibit lower expression of BSCL2-201 transcript compared to c.985C>T carriers (Sánchez-Iglesias et al., 2019), correlating with relatively milder neurological manifestations. In this case, the progression from regression to death was rapid, with the cause of death being SE. SE was common in PELD and always difficult to treat, having been the cause of death in at least four reported cases (Guillén-Navarro et al., 2013; Alaei et al., 2016; Fernández-Marmiesse et al., 2019).

Epilepsy was common in PELD, affecting 20 out of 24 patients. Among these, myoclonic seizures were the most common type. The high expression of the BSCL2 gene in the brain and the widespread deposition of aberrant protein may contribute to this phenomenon. The presence of myoclonic seizures, ataxia, and progressive neurological deterioration in this patient supports the diagnosis of Progressive Myoclonus Epilepsy (PME). This finding strengthens the evidence for the coexistence of PME and BSCL2 mutations, as previously suggested in the literature (Zhang et al., 2019; Opri et al., 2016; Ferranti et al., 2020; Serino et al., 2019). In the literature review, we found that PME is underdiagnosed in PELD. Therefore, we propose that PELD should be considered as one of the PMEs. The BSCL2 gene should be considered in gene panels for PME diagnostics.

All patients with 974dupG, 1048C>T, and 1076dupC variants displayed a lipodystrophic appearance. In contrast, those with the 445C>G and 566T>A heterozygous variants did not, while the phenotype for patients with the 985C>T variant remains uncertain. This suggests a potential link between genotype and lipodystrophic appearance. Given the small number of cases, this link still needs further investigation. We found that both compound heterozygotes and homozygotes may or may not display a lipodystrophic appearance, indicating that these features are independent of zygosity type, which is inconsistent with previous studies (Guillén-Navarro et al., 2013).

It has been reported that leptin-replacement therapy delayed neurological regression or allowed better seizure control in patients with PELD (Sánchez-Iglesias et al., 2019; Pedicelli et al., 2020; Araújo-Vilar et al., 2018). Unfortunately, the patient died 23 days after genetic cause confirmation, highlighting the importance of early diagnosis. Early genetic testing and diagnosis are crucial for improving outcomes in rare neurodegenerative disorders like PELD.

## Data Availability

The original contributions presented in the study are included in the article/supplementary material, further inquiries can be directed to the corresponding author/s.
